# HIV replication is associated to inflammasomes activation, IL-1β, IL-18 and caspase-1 expression in GALT and peripheral blood

**DOI:** 10.1371/journal.pone.0192845

**Published:** 2018-04-19

**Authors:** Manuel Gerónimo Feria, Natalia Andrea Taborda, Juan C. Hernandez, Maria Teresa Rugeles

**Affiliations:** 1 Grupo Inmunovirología, Facultad de medicina, Universidad de Antioquia UdeA, Medellín, Colombia; 2 Grupo de Investigaciones Biomédicas Uniremington, Programa de Medicina, Facultad de Ciencias de la Salud, Corporación Universitaria Remington, Medellín, Colombia; 3 Infettare, Facultad de Medicina, Universidad Cooperativa de Colombia, Medellín, Colombia; Public Library of Science, UNITED KINGDOM

## Abstract

**Background:**

Human immunodeficiency virus (HIV) promotes an inflammatory process, leading to the progressive loss of the functional capacity of the immune system. The HIV infection induces alterations in several tissues, but mainly in the gut-associated lymphoid tissue (GALT). However, the degree of GALT deterioration varies among infected individuals. In fact, it has been shown that HIV-controllers, who spontaneously control viral replication, exhibit a lower inflammatory response, and a relative normal frequency and function of most of the immune cells. Inflammasomes are molecular complexes involved in the inflammatory response, being NLRP1, NLRP3, NLRC4, AIM2 and Pyrin inflammasomes, the best characterized so far. These complexes regulate the maturation of cytokines of the IL-1 family, including IL-1β and IL-18. These cytokines have been associated with immune activation and expansion of HIV target cells, promoting viral replication. Interesting, some reports indicate that HIV induces the activation of the NLRP3 inflammasome, but the role of this, and other inflammasomes during HIV infection, especially in GALT, remains unclear.

**Objective:**

To compare the relative expression of inflammasome components and the proinflammatory response related to their activity, between HIV-progressors and HIV-controllers.

**Methods:**

GALT biopsies and peripheral blood mononuclear cells (PBMCs) from 15 HIV-controllers and 15 HIV-progressors were obtained. The relative expression of the following inflammasome components were evaluated by RT-PCR: NLRP3, NLRC4, NLRP1, AIM2, ASC, Caspase-1, IL-1β and IL-18. In addition, plasma concentration of IL-18 was evaluated as an indicator of baseline proinflammatory status. Finally, in supernatants of PBMCs *in vitro* stimulated with inflammasome agonists, the concentrations of IL-1β and IL-18 were quantified by ELISA.

**Results:**

HIV-progressors exhibited higher expression of IL-1β, IL-18 and caspase-1 genes in GALT and PBMCs compared with HIV-controllers. In addition, HIV-progressors had also increased expression of ASC in PBMCs. When plasma levels were evaluated, IL-18 was increased in HIV-progressors. Interesting, these patients also showed an increased production of IL-1β in supernatants of PBMCs stimulated *in vitro* with the agonists of AIM2, NLRP1 and NLRC4 inflammasomes. Finally, the expression of caspase-1, NLRP1, IL-1β and IL-18 in GALT or peripheral blood was correlated with CD4+ T-cell count and viral load.

**Conclusion:**

Our results suggest that during HIV-infection, the required signals to induce the expression of different components of the inflammasomes are produced, both in GALT and in periphery. The activation of these molecular complexes could increase the number of target cells, favoring HIV replication and cell death, promoting the disease progression.

## Introduction

The pathogenesis of HIV involves a complex interaction between several viral and host factors. So far, it is recognized that the gut-associated lymphoid tissue (GALT) is the main affected organ, as a consequence of the massive elimination of CD4^+^ T-cells, in particular Th17 cells that play an essential role in the mucosa homeostasis [1, 2]. The elimination of these and other cells such as enterocytes [3–5], induces structural damage in GALT, allowing microbial translocation from the intestinal lumen to systemic circulation, promoting excessive immune activation [6]. This state, currently recognize as the main immunopathogenic mechanism during HIV infection, is established during the acute phase and remains throughout the course of the infection, leading to cell exhaustion and activation-induced apoptosis [7]. Myeloid cells, including monocytes/macrophages and dendritic cells, are also affected by immune activation, as there is an increased level of microbial ligands recognized by Toll (TLR) and NOD (NLR) receptors inducing activation of NF-κB and the gene expression of proinflammatory cytokines such as IL-1β and IL-18 [8, 9]. In addition, endosomal TLR7 and TLR8 could detect HIV, increasing the inflammatory response [10, 11]. IL-1β and IL-18 are involved in the differentiation of naïve CD4^+^ T cells to Th1 and Th17 profiles [12–14], increasing even more the immune activation, and promoting viral replication since these cells are viral targets; in fact, these cytokines are increased in plasma of infected patients [15, 16], suggesting their involvement in HIV pathogenesis [10, 11].

Several molecules participate during the inflammatory process, including the large multimolecular complexes, so-called inflammasomes. These molecules activate caspase-1 and cytokines of the IL-1 family, including IL-1β and IL-18 [17]. Although several inflammasomes have been described, the best characterized are the nucleotide-binding domain leucine-rich repeat-containing (NLR) family (NLRP1, NLRP3, and NLRC4); the protein absent in melanoma 2 (AIM2), belonging to the PYHIN protein family; and the recently described Pyrin [18].

The excessive response of inflammasomes has been associated with some chronic inflammatory diseases such as type 2 diabetes, asthma, atherosclerosis, kidney disease and autoimmune disorders [19, 20]. In the context of HIV, some studies have found that HIV induces NLRP3 inflammasome activation as an alternative mechanism to maintain the inflammatory process. In fact, it has been previously published that HIV is able to induce the primary signal for NLRP3 inflammasome activation in macrophages [15]. In addition, other investigations have reported that dendritic cells exhibit high mRNA expression of NLRP3, IL-1β and caspase-1 in response to *in vitro* HIV infection, suggesting that this inflammasome might be involved in the pathogenesis of this infection [21, 22]. However, the role of these molecular complexes, especially in GALT, is unclear. Therefore, in a cohort of HIV-progressors and HIV-controllers, we compared the relative expression of inflammasome components and the proinflammatory response, related to their activity. The results were correlated with viral load and CD4^+^ T-cell count in both, peripheral blood and GALT, in order to determine the potential association between inflammasome activation and AIDS progression.

## Materials and methods

### Study population

Fifteen HIV-controllers, based on previously defined criteria [23, 24] and 15 HIV-progressors with viral loads between 10,000 and 100,000 copies/mL and CD4^+^ T-cell count >350 cells/μL, were included. Peripheral blood samples from all donors and GALT samples from 11 HIV-controllers and 13 HIV-progressors were obtained. All the enrolled individuals were antiretroviral therapy naïve and recruited from health insurance programs in Medellin, Colombia. The individuals enrolled signed a written informed consents prepared according to the colombian legislation, resolution 008430/1993 and approved by Ethical committee of the University of Antioquia (certificate 14-08-567) files.

### Viral load in plasma and CD4^+^ T-cell count

Plasma viral load was determined using the commercial assay RT-PCR Ampliprep-Cobas Amplicor (Roche, Indianapolis, IN; detection limit of 20 copies/mL), following the manufacturer’s protocol. The frequency of peripheral blood and GALT CD4^+^ T-cells was determined by flow cytometry. Briefly, peripheral blood was incubated with specific monoclonal antibodies at room temperature in the dark. The erythrocytes were lysed and the cells were washed twice with PBS and fixed with 2% paraformaldehyde. For GALT, cells were first incubated with IgG-blocking antibodies 20 μg/mL (eBioscience, San Diego, USA); then, cells were washed twice with PBS and incubated with specific monoclonal antibodies at room temperature in the dark. In this study, the following fluorescence labeled monoclonal antibodies were obtained from eBioscience (San Diego, USA): CD3 (Anti-CD3-FITC; clone UCHT1), CD4 (Anti-CD4-APC; Clone: RPA-T4) and CD8 -PE, clone: RPA-T8). The lymphocyte region was selected by "Size (SSC) vs. Forward (FSC) light scatter" parameters. CD8^+^ and CD4^+^ T-cells were selected from the CD3^+^ gate. The acquisition was performed on the FACS CANTO-II (BD) cytometer, using the software BD FACSDiva version 6.1.2.

### Isolation of mononuclear cells from GALT biopsies

Rectosigmoidoscopy and biopsies were performed as previously reported [25], using a flexible sigmoidoscope with single-use biopsy forceps FB-24K-1 (Olympus America Corp, Melville, NY, USA); from each subject, tissue samples were obtained from the rectum at 10 cm from anal verge. Four fragments were digested with collagenase type II from *Clostridium histolyticum* (Sigma; 0.5 mg/mL) diluted in RPMI 1640% and 7.5% fetal bovine serum (FBS) plus 100 U/mL penicillin and 100 mg/mL streptomycin (Gibco-BRL, Grand Island, NY) for 30 minutes at 37°C with shaking. After collagenase digestion, biopsy fragments were further disrupted by repeated passage through a syringe with a 16-gauge blunt-end needle (Stem Cell Technologies, Vancouver, BC, Canada). Rectal cells (RCs) were isolated from the fragments by passage through a 70 mM nylon strainer (Falcon, Lincoln Park, NJ). RCs were washed with PBS (Sigma-Aldrich, San Luis, MO) to remove excess of collagenase. Subjects with nodular lymphoid hyperplasia, ulcers, diverticulitis, adenoma and other benign or malignant growths were excluded from the study.

### *In vitro* activation of inflammasomes

Peripheral blood mononuclear cells (PBMCs) were isolated using Ficoll-hypaque gradient. Then, 1x10^5^ PBMCs were primed for 2 hours with 50 pg/mL ultrapure lipopolysaccharide (LPS) from *Escherichia coli*. The second activation signal was induced with specific inflammasome agonists, including ATP (2mM) for NLRP3; flagellin of *Salmonella typhimurium* (500 ng/mL) for NLRC4; poly(dA;dT) (50 μg/mL) for AIM2; and Muramyl dipeptide (MDP, 0.1 μg/mL) for NLRP1. After four hours of incubation, or 2 hours for ATP-treated PBMCs, the supernatants were harvested. All the agonists used were from Invivogen (California, USA). To minimize the effects of variability among individuals, the results were normalized according to the primed control cells (cells treated only with 50 pg/mL LPS), and expressed as fold increases.

### ELISA

The IL-1β production was quantified following the recommendations of the manufacturer of the OptEIA ™ Set commercial kit (BD Biosciences, San Diego, USA); for IL-18, the Human IL-18 Matched Antibody Pairs BMS267/2MST kit (eBioscience, Vienna, Austria) was used. IL-1β and IL-18 detection was performed in plasma of HIV infected individuals, and in supernatants of agonists-stimulated PBMCs.

### Gene expression of inflammasome components

To determine the transcriptional expression of genes associated with the inflammasomes (NLRP3, NLRP1, NLRC4, NLRP6, AIM2, ASC, and caspase-1) and their products (IL-1β and IL-18), total RNA extraction from PBMCs and GALT biopsies was performed using the RNeasy Mini Kit (QIAGEN, Inc., Valencia, CA, USA), following the manufacturer's instructions. RNA was treated with DNAse (DNase I, RNase-free, Qiagen, Hilden, Germany) and the cDNA was synthesized using the RevertAid H Minus First Strand cDNA Synthesis Kit (Thermo scientific, Waltham, Massachusetts, USA).

Gene expression was quantified by real-time PCR, using Maxima SYBR Green/ROX qPCR Master Mix (Thermo scientific, Waltham, Massachusetts, USA), specific oligonucleotides (**S1 Table**) and 2 μL cDNA of each sample in a final volume of 20 μL. A melting curve was included to confirm the specificity of the PCR product. All real-time PCR amplifications were performed using the CFX96 real-time system and data analysis using the software CFX Manager Version 1.5.534.0511 (Bio-Rad,Hercules, CA). Relative expression was calculated using ubiquitin to normalize the RNA content and the ΔCt method (1,8 ^(ct(target gene)-ct(ubiquitin gene)^) was applied. The same amount de cDNA was used in all cases [26].

### Statistical analysis

To compare data from HIV-controllers vs. HIV-progressors, Mann–Whitney U–2-tailed test was used. A *p* value <0.05 was considered statistically significant. Correlation analyses were based on Spearman correlation coefficient calculations. The statistical tests were performed using the GraphPad Software version 7.02.

## Results

### Prolonged control of viral infection in HIV-controllers

Demographic characteristics of HIV-infected donors are shown in **Table 1**. No significant differences were observed in the nadir CD4^+^ T cells between HIV- controllers and HIV-progressors. However, HIV-controllers exhibited lower viral loads and a higher CD4^+^ T cell count, compared with HIV-progressors. In addition, the mean time since HIV diagnosis was 62 months in HIV-controllers, evidencing their stable ability for exerting viral control; in fact, some of these individuals, could be classified as long-term non progressors.

**Table 1:** Demographic characteristics of HIV-infected donors.

	HIV-controllers (n = 15)	HIV-progressors (n = 15)	p-values*
**Age (years)**	29	27	0.1858
**Median (IQR)**	(24–39)	(29–32)	
**CD4^+^ T-cell count (cells/μL)**	769	400	0.0001
**Median (IQR)**	(642–996)	(344–633)	
**Nadir CD4^+^ T cells (cells/μL)**	601	466	0.05363
**Median (IQR)**	(335–1017)	(270–864)	
**Viral load copies/mL**	318	28801	0.0001
**Median (IQR)**	(162–1383)	(22105–63715)	
**Time after HIV-diagnosis (Months)**	62	42	0.0622
**Median (IQR)**	(20–162)	(12–62)	

IQR: Interquartile range

*Mann-Withney test

### Higher expression of IL-1β, IL-18 and caspase-1 genes in GALT from HIV-progressors

**Fig 1:**
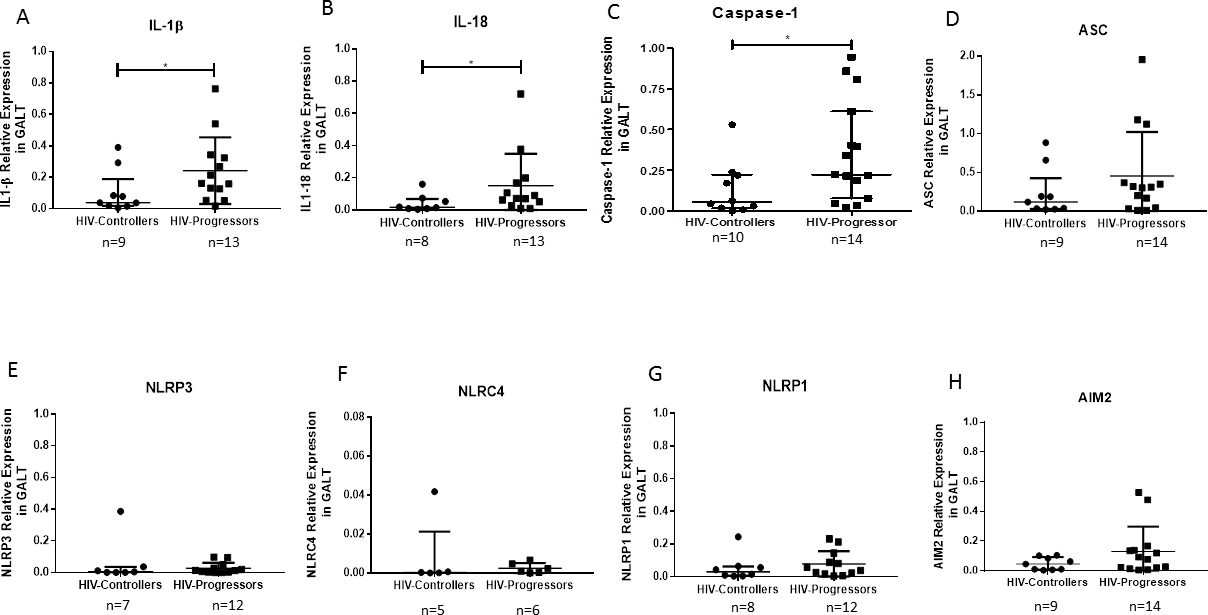
Relative expression of the inflammasomes components in GALT. The RNA levels of IL-1β (A), IL-18 (B), caspase- 1 (C), ASC (D), NLRP3 (E), NLRC4 (F), NLRP1 (G), and AIM2 (H) were quantified in GALT from HIV-1-controllers and HIV-1-progressors, by qPCR. The ubiquitin gene was used as constitutive gene to normalize the RNA content. Relative expression is referred to ubiquitin expression. The results are presented as medians and interquartile ranges. Statistical comparison was performed using a Mann-Whitney U test with a 95% confidence level. Significant differences are indicated at the top of the figure (* p <0.05).

Considering that GALT is the main affected tissue during HIV infection, the expression of the inflammasome components in this organ was evaluated. Compared to HIV-controllers, HIV-progressors had a higher expression of IL-1β (Fig 1A), IL-18 (Fig 1B) and caspase-1 (Fig 1C). In contrast, we did not observe any differences in the expression of other inflammasome components, including, ASC, NLRP3, NLRC4, NLRP1, and AIM2 (Fig 1D–1H), between groups.

### Increased expression of IL-1β, IL-18, caspase-1 and ASC genes in PBMCs from HIV-progressors

**Fig 2:**
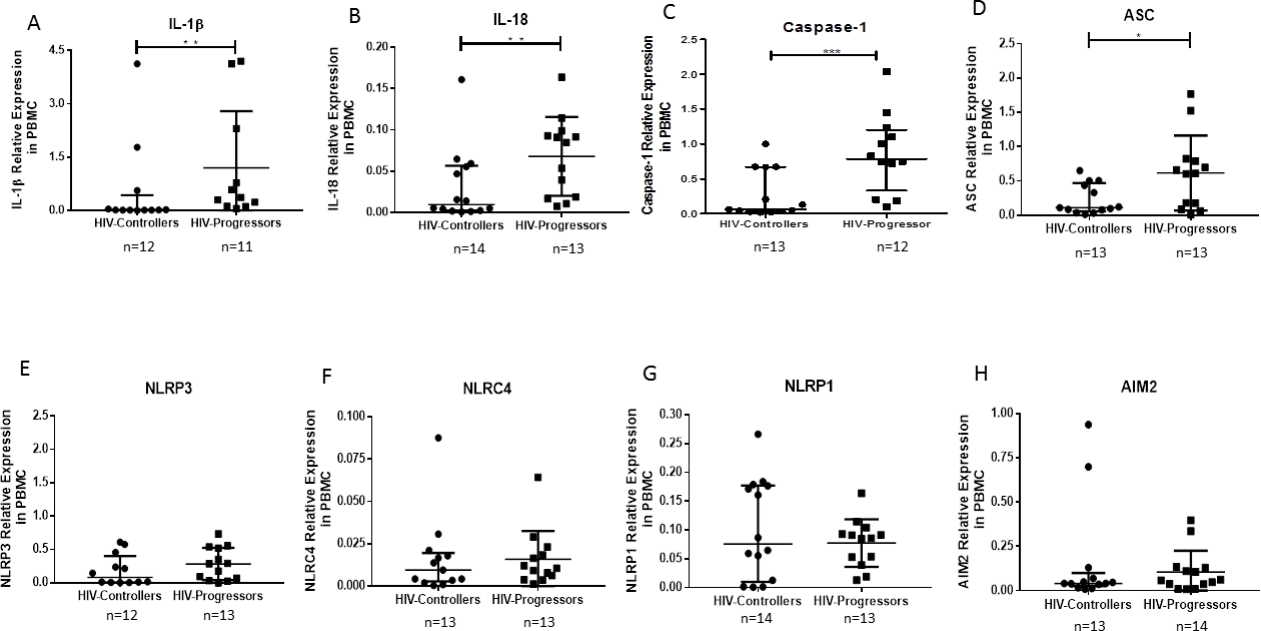
Relative expression of the inflammasomes components in PBMCs. The RNA levels of IL-1β (A), IL-18 (B), caspase- 1 (C), ASC (D), NLRP3 (E), NLRC4 (F), NLRP1 (G), and AIM2 (H) were quantified in PBMCs from HIV-1-controllers and HIV-1-progressors, by qPCR. The ubiquitin gene was used as constitutive gene to normalize the RNA content. Relative expression is referred to ubiquitin expression. The results are presented as medians and interquartile ranges. Statistical comparison was performed using a Mann-Whitney U test with a 95% confidence level. Significant differences are indicated at the top of the figure (* p <0.05) (**p<0.01) (***p<0,001).

The relative expression of IL-1β (Fig 2A), IL-18 (Fig 2B), caspase-1 (Fig 2C) and ASC (Fig 2D) genes was higher in PBMCs from HIV-progressors than HIV-controllers. No significant differences were observed in other inflammasome components, including NLRP3, NLRC4, NLRP1, AIM2 (Fig 2E–2H).

### Higher levels of IL-18 in plasma from HIV-progressors

**Fig 3:**
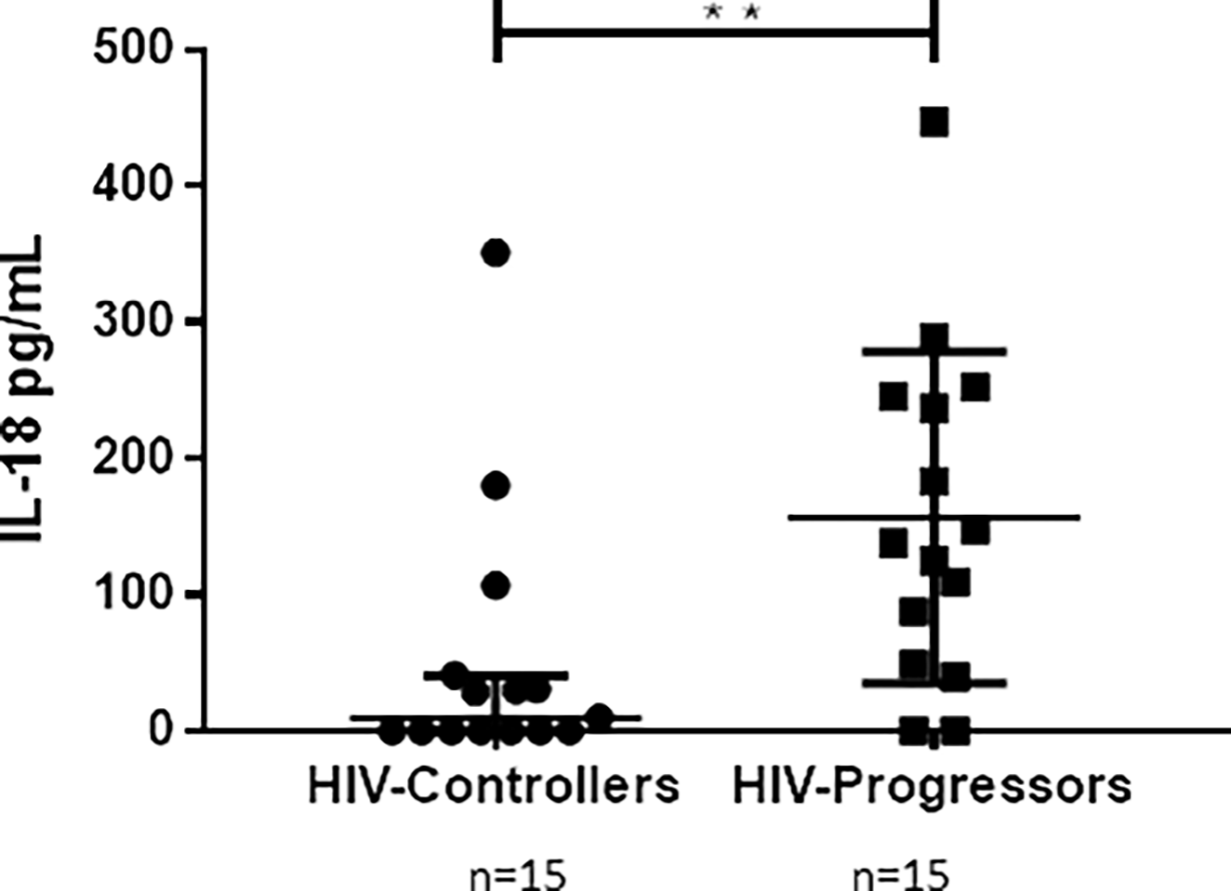
Plasma levels of IL-18 in HIV-1-infected donors. IL-18 was quantified in plasma samples by ELISA. The results are presented as median and interquartile ranges. Statistical comparison between groups was performed using a Mann-Whitney U test with a confidence level of 95%. Significant differences are indicated at the top of the figure (** p <0.01).

In order to determine the basal inflammatory status associated with inflammasomes activity, IL-18 was quantified in plasma from HIV-infected donors. HIV-progressors have a higher IL-18 concentration compared to HIV-controllers (131.6 pg/mL vs. 4.8 pg/mL, Fig 3).

### HIV-progressors exhibit increased production of IL-1β through AIM2, NLRP1 and NLRC4 inflammasomes, in an *in vitro* model

**Fig 4:**
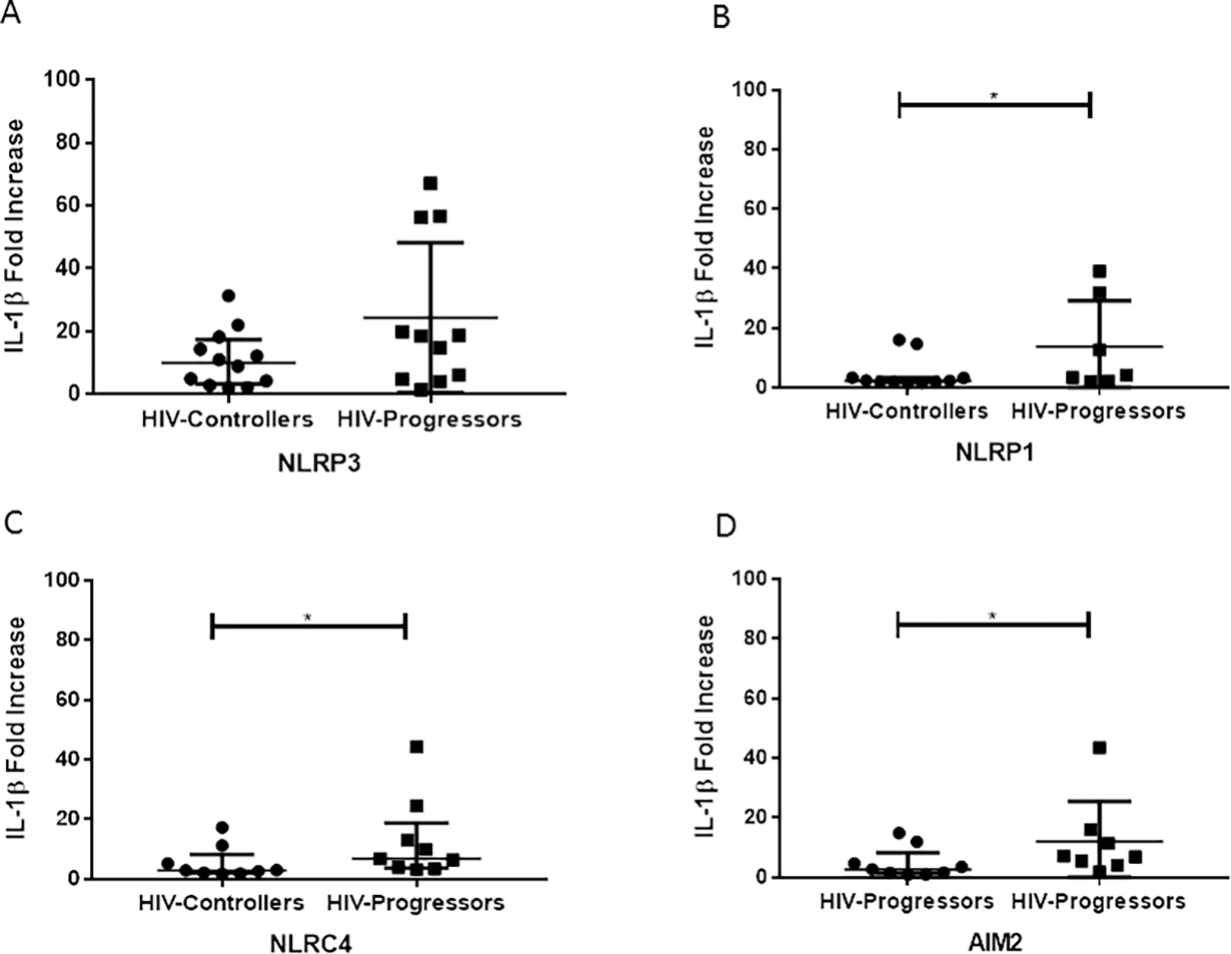
Activation of inflammasomes in PBMCs from HIV-1-infected donors. LPS-primed PBMCs were treated with inflammasomes agonists (ATP for NLRP3 (A), MDP for NLRP1 (B), Flagellin for NLRC4 (C) and poly(dA:dT) for AIM2 (D), and the IL-1β release was quantified by ELISA in the supernatants. Fold increase is referred to primed cells (cells treated only with 50 pg/mL LPS). The results are presented as median and interquartile ranges. Statistical comparison between groups was performed using a Mann-Whitney U test with a confidence level of 95%. Significant differences are indicated at the top of the figure (*p <0.05).

To determine the specific inflammasomes associated to HIV progression, through the IL-1β and IL-18 production, an *in vitro* assay was performed. A higher production of IL-1β in PBMCs from HIV-progressors compared to HIV-controllers, was observed when the cells were treated with agonist of NLRP1 (Fig 4B), NLRC4 (Fig 4C) and AIM2 (Fig 4D) inflammasomes. In the case of the NLRP3 inflammasome (Fig 4A), there were no significant differences in IL-1β release between both groups of patients.

### The expression of caspase-1, NLRP1, IL-1β and IL-18 in GALT or peripheral blood correlates with CD4^+^ T cell count and viral load

**Fig 5:**
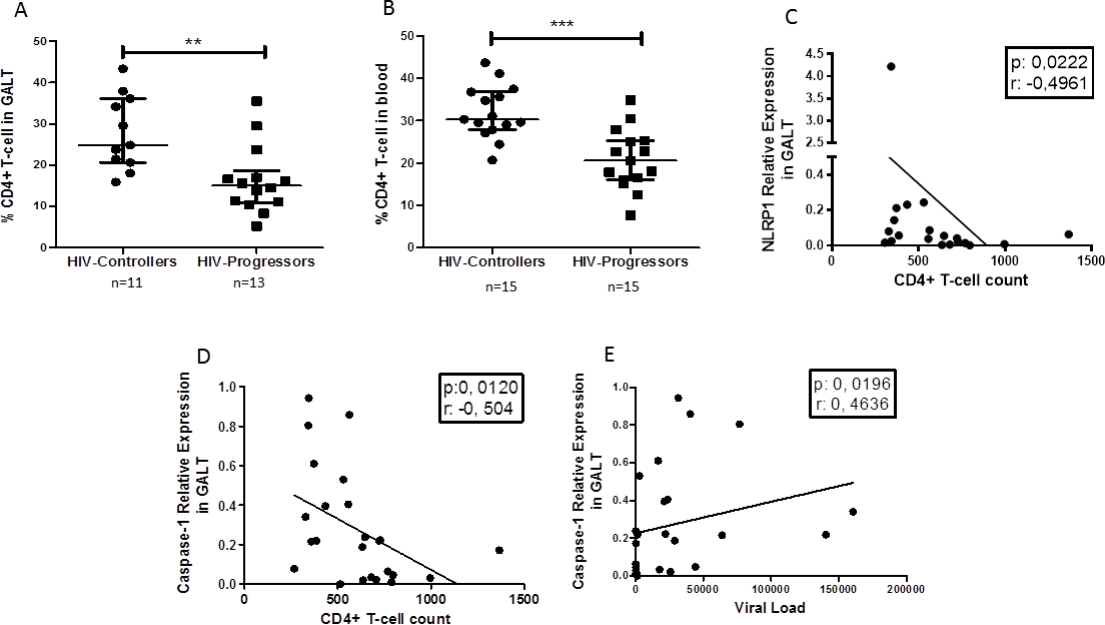
Correlation between the expressions of the inflammasome components in GALT with the CD4^+^ T-cell count and viral load. Higher frequency of CD4^+^-T cells in GALT (A) and peripheral blood (B) in HIV-controllers compared to HIV-progressors. The relative expression of NLRP1 in GALT was negatively correlated with the CD4^+^ T-cell in peripheral blood (C). Equally, the relative expression of caspase-1 was negatively correlated with the CD4^+^ T cells in peripheral blood (D) and positively with the viral load (E). Statistical comparison between groups was performed using a Mann-Whitney U test with a confidence level of 95%. Significant differences are indicated at the top of the figure (**p <0.01) (***p<0,001). The correlations were performed with a Spearman test. The r value and the p-value of the correlations are indicated at the top of each figure; a p-value less than 0.05 was considered as a significant correlation.

Initially, we compared the frequency of CD4^+^ T cells in GALT and peripheral blood between the HIV-infected donors. As expected, HIV-controllers exhibit higher frequency of CD4^+^ T-cells in GALT (Fig 5A) and peripheral blood (Fig 5B). Then, in order to determine if the altered expression of inflammasome components and their products were involved in HIV progression, a correlation analysis was performed. In GALT, the relative expression of NLRP1 (Fig 5C) was negatively correlated with CD4^+^ T-cell count. Likewise, caspase-1 was negatively correlated with CD4^+^ T-cell count (Fig 5D) and positively correlated with viral load (Fig 5E).

**Fig 6:**
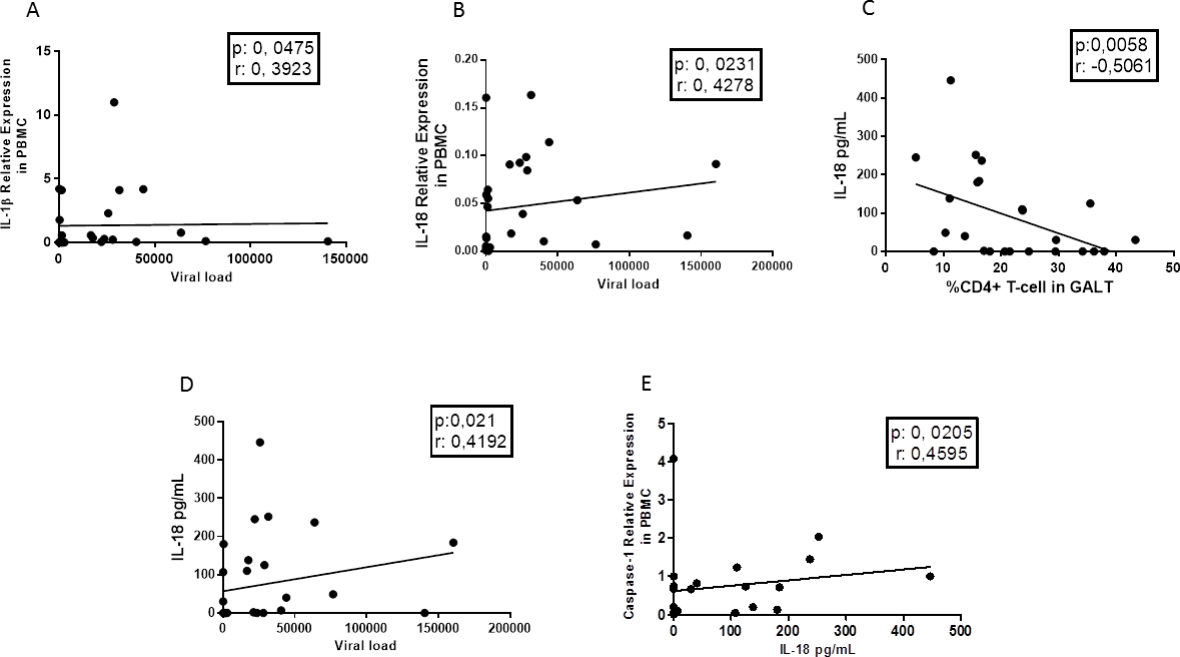
Correlation between the expression of the inflammasome components and products in peripheral with the CD4^+^ T-cell count and the viral load. The relative expression of IL-1β and IL-18 in PBMCs was positively correlated with viral load (A) (B). The plasma concentration of IL-18 was negatively correlated with the CD4^+^ T-cell frequency in GALT (C) and positively with the viral load (D). The expression of caspase-1 was positively correlated with plasma concentration of IL-18 (E). The correlations were performed with the Spearman test. The r value and the p-value of the correlations are indicated at the top of each figure; a p-value less than 0.05 was considered as a significant correlation.

Finally, in peripheral blood, viral load was positively correlated with the relative expression of IL-1β and IL-18 (Fig 6A and 6B). Interestingly, the protein level of IL-18 was positively correlated with viral load and negatively correlated with the frequency of CD4^+^ T cells in GALT (Fig 6C and 6D). Finally, the relative expression of caspase-1 and the protein level of IL-18 were positively correlated (Fig 6E).

## Discussion

HIV is characterized by an unregulated inflammatory response and the consequent immune exhaustion [27]. Inflammasomes could be one of the immune components involved in this process. They are important in the regulation of the caspase-1 activity to induce the proteolytic maturation of the IL-1β and IL-18. These proinflammatory cytokines are particularly increased in HIV infected individuals, suggesting their role in AIDS progression [15, 28]. In fact, it has been shown that HIV has the ability to activate the NLRP3 inflammasome [15]; however, the definite role of this, and other inflammasomes during the pathogenesis of HIV is still unclear. Here, we evaluated the role of different inflammasomes in the pathogenesis of this infection, by studying two different groups of infected patients: HIV-controllers and HIV-progressors.

We observed that HIV-progressors exhibit higher relative expression of IL-1β, IL-18 and caspase-1 in GALT, and in PBMCs, compared with HIV-controllers. In addition, HIV-progressors had also increased expression of ASC in PBMCs, which could reflect the fact that this protein is mainly expressed by monocytes and lymphocytes [29]. In relation to our findings, *ex vivo* studies have demonstrated that HIV induces the expression of caspase-1, NLRP3, IL-1β and IL-18 in cerebral white matter and microglial cells [30, 31], promoting an inflammatory process at the central nervous system, and HIV-associated neurocognitive disorders [32]. In addition, *in vitro* HIV-infected dendritic cells and monocytes-derived macrophages have shown increased expression of others inflammasome components such as NLRP3, ASC and AIM2 [21, 22, 33]. Similar results have been observed in primary cells or HIV infected cell lines [34, 35], indicating the ability of the virus for inducing the priming signal for the activation of the inflammasomes in different tissues, as one of the mechanisms responsible for the systemic hyperactivation observed in infected individuals, even in those under highly active antiretroviral therapy (HAART) [36].

It is clear now that immune activation is established early during the acute phase of the infection, leading to diverse alterations in GALT, favoring viral replication and eventually, immune exhaustion [37]. Transcriptome studies in simian immunodeficiency virus (SIV)-infected monkeys have shown that several proinflammatory genes are upregulated, including those coding for various inflammasome components such as NLRs, TLRs, and caspases [38]. Supporting this evidence, here we show that HIV-progressors had a higher relative expression of IL-1β, IL-18 and caspase-1, compared with HIV-controllers in GALT and PBMCs samples. These findings might suggest that the inflammasomes might indeed influence the clinical course of the HIV infection, by participating in the inflammatory process, recognized as one of the main pathogenic mechanisms associated to AIDS progression. In fact, the massive elimination, in GALT, of HIV non-permissive cells occur by pyroptosis, which is induced mainly by the activation of the caspase-1. Interestingly, a higher expression of caspase-1 in both, PBMCs and GALT samples was observed in HIV-progressors compared with HIV controllers. In addition, the relative expression of the caspase-1 in GALT had a negative and a positive correlation with CD4^+^ T-cell count and with load viral, respectively. These results support the potential role of the caspasa-1 in promoting immune deterioration during HIV infection and viral replication.

The lack of differences between both study groups in the relative expression of the PRRs, NLRP3, NLRC4, NLRP1 and AIM2 might indicate that these proteins do not influence HIV progression, although the impact of the low sample size cannot be ruled-out. Similar results were reported in studies comparing HIV infected patients with uninfected individuals [31], where a higher expression of IL-1β, IL-18, and caspase-1 were found in cerebral white matter from HIV- infected individuals.

Another characteristic of HIV-infected individuals is the expression of high levels of the proinflammatory cytokines IL-1β and IL-18 [15, 39]. In fact, we observed that HIV-progressors had higher levels of circulating IL-18 compared with HIV-controllers. During HIV infection, low plasma concentrations of the IL-18-binding protein (which modulate the proinflammatory activity of the IL-18) have been reported [40]; this finding might contribute to the exacerbated inflammatory process that characterizes HIV-progressors [25]. It is important to note that IL-18 is involved in the differentiation of naïve T cells to Th1 and Th17 cells [13, 14, 41, 42], which are highly susceptible to HIV infection; therefore, high levels of this cytokine could promote massive viral replication, immune activation and the consequent mucosal damage. Exploring the frequencies of different CD4+ T cell subpopulations in HIV patients, exhibiting different progression patterns, could support or ruled-out this hypothesis.

Unfortunately, we did not detect IL-1β, although it is also processed by the inflammasome. The presence of IL-1β inhibitors in the plasma samples is the most likely explanation for this result, since exogenous addition of this cytokine to the plasma did not result in the subsequent detection of this protein. In fact, higher levels of the IL-1RII, natural inhibitor of this cytokine, have been previously reported in HIV-infected [43]. Since previous reports have indeed indicated the presence of high levels of this cytokine in HIV infected individuals, further studies evaluating its correlation with progression are required.

Previously, *in vitro* studies demonstrated that HIV or its products (viral proteins Vpr and Tat or the non-integrated viral cDNA) activates NLRP3 and AIM2 inflammasomes [15, 30, 35, 44, 45]. However, the inflammasomes most likely responsible for the high production of IL-1β and IL-18 are still unclear. In this study, we observed that HIV-progressors produce increased levels of IL-1β through AIM2, NLRC4 and NLRP1 inflammasomes, in response to the specific agonists. In contrast to other studies, in cell cultures we did not detect IL-18 [46]. It seems that the level of production of this cytokine in cultures is very low, even below the detection limit of the commercial kit used; in fact, in previous studies, the protein had to be concentrated for the ELISA determination [47].

In relation with the higher production of IL-1β through the AIM2 inflammasome observed in HIV-progressors, it has been previously demonstrated that the proteins of the HIN200 family, AIM2 and IFI16 (interferon-γ inducible protein 16) interact with the HIV cDNA present in the cytoplasm of non-permissive cells. This interaction results in caspase-1 activation, which in turns induce pyroptosis, promoting the structural and functional deterioration of GALT [44, 45]

To our knowledge, this is the first report indicating a higher production of IL-1β through the NLRC4 and NLRP1 inflammasomes during HIV infection, mainly in HIV-progressors. However, it has been demonstrated that the activation of both inflammasomes can result from the interaction with the bacterial proteins flagellina and T3SS for the NLRC4 inflammasome [48], and MDP for the NLRP1 inflammasome [49]. Particularly, flagellina, T3SS and MDP are proteins found in the gut microbiote [50, 51] that could be translocated to systemic circulation during HIV infection because of the increased permeability of the GALT mucosa, previously reported in infected patients [52]. In the opposite side, in HIV-controllers, the low levels of IL-1β and IL-18 could be associated with the effectiveness of regulatory mechanisms, such as post-translation modifications of inflammasome proteins, including deubiquitination of NLRP3 or phosphorylation of ASC proteins [53, 54].

In relation with the NLRP3 inflammasome, no differences were observed between HIV groups. These results might correspond to the fact that the NRLP3 inflammasome has multiple agonists, such as ROS and ATP that could induce its activation through different intracellular pathways. In this sense, the physiopathology experienced by HIV-infected individuals, independent of their ability to control viral replication, induces the production of several DAMPs responsible for the activation of the NLRP3 inflammasome.

Finally, the results suggest that during HIV-infection the required signals to induce the relative expression of different components of the inflammasome are produced, both in GALT and in periphery. The activation of the molecular complexes increased the number of target cells, favoring HIV replication and cell death (by different mechanisms, including pyroptosis), promoting the development of disease.

## Supporting information

S1 Table Primers list. Sequences used for mRNA amplification of inflammasome-related genes.
(DOCX)
