# Supplementary material for: HIV replication is associated to inflammasomes activation, IL-1β, IL-18 and caspase-1 expression in GALT and peripheral blood
Source: PLoS One. 2018 Apr 19;13(4):e0192845. doi: 10.1371/journal.pone.0192845 (PMC5909617; doi:10.1371/journal.pone.0192845)
Supplement: S1 Table — Sequences used for mRNA amplification of inflammasome-related genes. (DOCX) [file pone.0192845.s001.docx]

**Supporting information**

| **Gen** | **Sequence of primers 5'-3'** | **Annealing temperature** | **Product length (bp)** |
| --- | --- | --- | --- |
| **IL-1β** | Fw: CTTTGCCGATCCGCCGC | 60 °C | 174 |
|  | Rv: ATCACGCCCTGGTGCCTGG |  |  |
| **IL-18** | Fw: ATGGCTGCTGAACCAGTAGAAG | 62 °C | 292 |
|  | Rv: CAGCCATACCTCTAGGCTGGC |  |  |
| **NLRP3** | Fw: AGCACCAGCCAGAGTCTAAC | 57 °C | 123 |
|  | Rv: CCCCAACCACAATCTCCGAAT |  |  |
| **NLRP1** | Fw: CTATACTTCCCGAGGCATCCTT | 56 °C | 301 |
|  | Rv: GGTCTTGGAAGTCAGTGTGAGT |  |  |
| **NLRC4** | Fw: CTCTCATGGTGGAAGCCAGTCC | 56 °C | 301 |
|  | Rv: ACAGAGACTTGACTATGTAATCC |  |  |
| **AIM2** | Fw: AAGCGCTGTTTGCCAGTTAT | 55 °C | 231 |
|  | Rv: CACACGTGAGGCGCTATTTA |  |  |
| **ASC** | Fw: AACCCAAGCAAGATGCGGAAG | 62 °C | 82 |
|  | Rv: TTAGGGCCTGGAGGAGCAAG |  |  |
| **Caspase-1** | Fw: CAAGGGTGCTGAACAAGG | 60 °C | 278 |
|  | Rv: GGGCATAGCTGGGTTGTC |  |  |
| **Ubiquitin** | Fw: CCTTCAAACCACCTAAGGTTGC | 58, 7 °C | 109 |
|  | Rv: GTGCTGGAGACCACTGTGATGG |  |  |

**S1Table. Primers list.** Sequences used for mRNA amplification of inflammasome-related genes.

Fw: Forward primer; RV: Reverse primer; bp: base pair
